# Supplementary material for: Are carbon emissions trading and green financial instruments synergistic? -Comprehensive quantitative research based on content analysis
Source: PLoS One. 2024 Mar 7;19(3):e0298601. doi: 10.1371/journal.pone.0298601 (PMC10919601; doi:10.1371/journal.pone.0298601)
Supplement: S1 Data — (ZIP) [file pone.0298601.s001.zip › Data/地级市Z_F工作报告.zip!/║■▒▒/╗╞╩»╩╨/└┤╫╘╣1⁄2╓┌║┼í╛┬φ┐╦╩2╛▌═°í┐_2015.docx]

各位代表：

现在，我代表市人民政府向大会报告工作，请予审议，并请市政协各位委员和其他列席人员提出意见。

一、2014年工作回顾

2014年，是我国全面深化改革的开局之年，也是我市发展经受新考验的一年。一年来，面对国际国内环境复杂多变、经济下行压力加大的严峻形势，在市委的正确领导下，在市人大、市政协的监督支持下，我们紧紧依靠全市人民，深入实施“生态立市、产业强市，加快建成鄂东特大城市”发展战略，积极应对，主动作为，竞进提质，乘势勇进，全市经济社会发展在新常态下实现难中有为、稳中向好。

——经济社会保持平稳较快发展。预计，全市完成生产总值1207亿元，增长8.7%；规模以上工业增加值592亿元，增长9%；固定资产投资1149.5亿元，增长21.3%；社会消费品零售总额519.7亿元，增长13.3%；外贸出口14.47亿美元，增长20.1%；地方公共财政预算收入89.38亿元，增长14.1%；城镇常住居民人均可支配收入、农村常住居民人均可支配收入分别增长9.5%、12%；城镇登记失业率2.26；人口自然增长率8.43‰。在全国资源枯竭城市转型首次年度绩效考评中，我市被评为优秀等级城市。连续十年获得全省金融信用市。大冶实现百强再进位。

——产业转型升级步伐加快。深入实施“两计划两工程”，新增规模以上工业企业131家，高端装备制造业集群入选省级重点。加快传统产业改造升级，淘汰落后产能7.95万吨，关停“五小”企业367家。成功获批全国工业绿色转型发展、国家信息消费、全国内贸流通改革和发展试点城市，黄金山工业新区被列为国家低碳工业园区试点。大力培育战略性新兴产业，电子信息产业增幅高达50以上。深入开展“增信心、解难题，服务工业企业”专项行动，解决企业难题188个。安排财政间歇资金5000万元扶持中小企业发展，新增中小企业担保贷款110亿元。狠抓服务业提档升级，商贸流通市场主体培育取得新突破，武商购物中心、摩尔城等项目建成开业，万达广场等项目顺利推进，苏宁易购等知名品牌签约落户，中小商贸流通企业服务平台建成运营，第三产业占比提高1.3个百分点。东方山景区成功晋级4A 落霞小说。狠抓农业增产增效，新增农民合作社487家，其中国家级示范社5家；新增农产品加工规上企业12家、国家级休闲农业示范点1家、家庭农场158家。狠抓创新驱动，中科院黄石育成中心挂牌成立，新增院士工作站1家、省工程实验室3家，完成发明专利申请量928件，新认定高新技术企业17家，一批企业分别荣获国家、省科技进步奖励，新增中国驰名商标1件、省著名商标36件。高新技术产业增加值增长9。我市获批创建全国质量强市示范城市。

——对外开放水平明显提升。强力推进产业链招商和项目建设，成功引进欣兴电子等33个总投资430亿元的电子信息产业项目，实施亿元以上项目432个，沪士电子一期等242个项目竣工投产，华纳铜箔等217个项目开工建设，光谷产业园等项目加快推进。加快开放平台建设，大冶湖生态新区设施进一步完善，汪仁污水处理厂建成使用，棋盘洲码头一期工程完工，棋盘洲保税物流中心通过国家验收，做到当年申报、当年建成、当年通关，黄石成为全省第一家拥有保税物流中心的地级市。大力推动对外投资和经济合作，新增2家境外投资企业，外派各类劳务3200多人次，华新水泥在塔吉克斯坦投资的项目，成为“丝绸之路经济带”建设的一大亮点。

——生态环境建设成效明显。统筹推进“五城同创”，大力实施“五边三化”、“八园六带”工程，关停露天矿山83家，治理工业企业重点污染源460家，完成植树造林和“五边”区域绿化14万亩，新增和改造城区绿地125公顷，完成181个自然村村庄整治，新增国家级绿色矿山2家。大力开展“治气、治水、治土”专项行动，国家二级标准以上天数达315天，亚行贷款水污染综合治理项目稳步实施。强力推进节能减排，一批节能技改项目建成投产，全市水泥行业实现全面脱硝，万元生产总值能耗和主要污染物减排全面完成省定目标，其中万元生产总值能耗提前一年完成“十二五”规划目标。加大环境执法力度，依法查处环境违法案件64件。我市被列为全国第四批餐厨废弃物资源化利用和无害化处理试点城市、全国首批创建新能源示范市。成功举办首届园博会，荣获湖北省首届园博会承办权。

——新型城镇化加速推进。城市总体规划修编已报国务院审批。编制完成生态立市产业强市总体规划、大冶湖生态新区核心区控制性详规、开发区“两镇一区”和陈贵镇“四化同步”规划。城乡一体化迈出坚实步伐，大冶湖生态新区16个重大基础设施项目全面启动。“三万”活动深入开展。实施“四化同步”示范村项目46个，5个乡镇被列为全国重点镇，3个村入选中国传统古村落。城镇化率比上年提高0.25个百分点。综合立体交通体系日趋完善，武黄城际铁路建成通车，花湖物流中心、罗桥物流园、有色物流园等建成使用；武九客运专线、棋盘洲长江大桥连接线开工建设。完成西河路、发展大道综合改造，城区主干道全面刷黑，新建、恢复停车场7处。新建和改造农村公路405公里。开展城市环境综合整治，新增省级卫生先进单位10家、卫生社区16个。开工各类水利工程2300多处，新增、改善灌溉面积21.45万亩，解决12.99万农村居民饮水不安全问题。实施土地整理项目11万亩。减少贫困人口1.3万人。

——重点领域改革全面深化。加快推进工商注册资本登记制度改革，推行“先照后证”、“三证联办”和企业信用信息公示制度，新登记各类市场主体45380户。对全市15类4291项行政权力及服务事项进行清理确认，将市级29个审批事项下放城区。建立健全公共资源交易管理机制。深化财政改革，实行全口径预算和绩效管理。积极推进市级投融资公司改革。汇波材料成功登陆“新三板”，凯程环保、九味食品实现股权交易挂牌，发行中小企业区域集优债3000万元。统筹推进其他领域改革，工矿废弃地复垦利用试点任务圆满完成，积极开展全国采矿权调查试点工作，大冶全面完成土地确权颁证工作。新增土地流转面积2万亩。一批国企改革遗留问题得到妥善解决。

——民生福祉进一步改善。各级财政用于民生支出增长14.5 。扎实抓好创业带就业工作，帮扶成功创业8220人，带动就业3.06万人，帮助困难人员再就业2.22万人。加强社会保障体系建设，全市社会保险参保总人数达247.59万人(次)，征缴社会保险费37.50亿元，城乡居民养老保险参保率达99.8，新农合参合率达99.75，城乡低保实现应保尽保。顺利完成企业退休人员基本养老金调标。开工建设保障性住房12607套，建成14030套，分配11100套，我市被列为全国共有产权住房试点城市。新建和改造63个乡镇中心幼儿园，投入2.86亿元改善义务教育薄弱学校办学条件，三个城区通过国家义务教育均衡发展评估验收。市群艺馆和公共文化数字服务平台全面建成，大冶荣获“全国文化先进县市”称号，华新文物保护项目获国家资金支持，建成一批农村文化礼堂，全市“三馆一站”免费开放。荣获省第十五届运动会承办权，成功举办全国乒乓球锦标赛、首届磁湖户外运动节等大型体育赛事活动，我市在省第十四届运动会上获金牌数列第三。金山老年公寓一期投入使用，市福利院新院开工建设。残疾人救助、就业服务取得新成效。加强和创新社会治理，加快构建市级社区公共服务综合信息平台，我市被列为全国社区治理和服务创新试验区，农村网格化建设稳步实施，深入推进平安黄石建设，加强矛盾纠纷调处工作，依法打击违法犯罪活动，群众安全感满意度明显提升。安全生产较大以上事故起数和死亡人数实现“双下降”。疾病防控和食品药品安全保障得到加强。政府十件实事全面完成。“十三五”规划编制和地理国情普查工作全面启动。加强“双拥”工作，支持军队建设，军政军民关系更加密切。与此同时，对台、保密、档案、妇女儿童、老龄、援藏、援疆、援利川、统计、外事、侨务、民族、宗教、国家安全、编制、科普、防震减灾、民防、气象、史志等各项工作都取得新的成绩。

——政府作风明显好转。扎实开展党的群众路线教育实践活动，认真落实中央“八项规定”、省委“六条意见”和市委“六点要求”，大力整治“四风”，全市公开和政风行风评议，认真做好政府新闻发布工作，政府施政更加公开透明。深入开展“六五”普法，法治黄石建设取得新成效。

各位代表，过去的一年，在这么困难的情况下，取得这样的成绩，实属不易，凝聚了全市人民的心血和汗水。借此机会，我代表市人民政府，向全市人民，向给予政府工作大力支持和有效监督的人大代表、政协委员，民主党派、工商联、无党派人士，各人民团体，向驻黄解放军、武警官兵，向所有关心支持黄石建设和发展的海内外人士，向在黄石投资的创业者和建设者，表示衷心的感谢!

在肯定成绩的同时，我们也清醒地看到，我市经济社会发展还面临一些困难和问题，主要是项目支撑不足，部分企业生产经营困难，经济下行压力较大；总量不大与结构矛盾相交织，传统产业受国家调控政策影响较大，新的增长点短期内难以显现，产业转型任重道远；节能减排、生态修复治理任务十分艰巨；少数干部抓工作的观念、方式方法还不能有效适应新常态，工作作风、效率有待进一步改进和提高，等等。这些困难和问题事关全局，我们必须引起高度重视，认真加以解决。二、2015年工作安排

今年，是全面深化改革的关键之年，是全面推进依法治国的开局之年，也是“十二五”规划的收官之年。在新常态下，黄石面临的形势依然错综复杂，但随着我国全面深化改革红利的不断释放，“一带一路”和长江经济带建设的深入推进，国家和省里实施定向精准调控，以及出台扩投资、促转型、优生态系列措施，将为我市新一轮发展提供多重叠加的重大机遇。我们必须增强机遇意识、进取意识、担当意识，以更大的力度攻改革之坚、克转型之难，奋力谱写发展新篇章。

政府工作的总体要求是：认真贯彻落实党的十八大、十八届三中、四中全会、习近平总书记系列重要讲话和三级经济工作会议精神，坚持稳中求进的工作总基调和“竞进提质、升级增效”的工作总要求，牢牢把握“市场、绿色、民生”三维纲要，深入实施“生态立市、产业强市，加快建成鄂东特大城市”发展战略，以提高经济发展质量和效益为主线，以法治政府建设为保障，大力度扩投资促增长，大力度调结构转方式，狠抓改革攻坚，突出创新驱动，统筹城乡发展，增进民生福祉，促进经济平稳较快发展和社会和谐稳定，全面完成“十二五”规划目标任务。

主要预期目标是：地区生产总值增长9.5%；规模以上工业增加值增长10%；固定资产投资增长20%以上；居民消费价格涨幅控制在3.5%以内；社会消费品零售总额增长13.2%；地方公共财政预算收入增长10%；外贸出口总额增长11%；城镇常住居民人均可支配收入增长9%；农村常住居民人均可支配收入增长10%；城镇登记失业率控制在4.5%以内；人口自然增长率控制在8.5‰以内。确保完成省下达的节能减排任务。

围绕上述要求和目标，主要抓好以下八个方面的工作：

(一)积极扩大投资，努力保持经济平稳较快增长

狠抓招商引资。进一步整合专业招商小分队力量，围绕电子信息、高端装备制造、智能模具等产业，通过在国内重点区域举办专题招商推介会，努力引进一批大项目好项目。开展精准招商，加强与跨国公司、央企、知名民营企业协作，引导投资新兴产业、新兴业态。坚持重落地、重投产，强化招商引资考核，加大招商项目后续服务和督查力度，切实提高项目的履约率、落地率和资金到位率。

狠抓项目建设。以省里开展的“项目建设竞进年、项目储备提质年、社会投资促进年”活动为契机，抓好501个亿元以上导向性项目的实施，确保全年固定资产投资完成1400亿元。优化投资结构，加大对工业投资的进度跟踪和督办协调力度，力争工业投资增长25以上。着眼“十三五”发展，认真策划储备一批事关黄石转型发展的重大项目，力争更多的项目进入国家、省“十三五”规划的笼子。推行重点项目建设直通车服务，优化项目建设环境。

狠抓民间投资。全面落实促进民营经济发展和扩大民间投资的政策措施。制定非公有制企业进入特许经营领域具体办法。策划一批市级示范项目鼓励社会投资。大力实施市场主体增量工程，落实结构性减税政策措施，支持民营企业通过上市、发行债券、引进股权投资等多渠道融资。支持黄石籍企业家回乡投资兴业。

(二)实施创新驱动战略，加快推进产业转型升级

加快培育新兴产业。坚持把电子信息、高端装备制造、智能模具作为产业转型的着力点和主攻方向，将电子信息产业作为省级重点产业来打造。筹措20亿元资金，支持重点产业发展。编制完成电子信息产业发展规划。支持沪士电子300万平方米印刷电路板、欣兴电子高密度互连积层板、PCB配套产业园等项目建设，促进PCB产业集群快速壮大。围绕华纳国际项目，引进一批电子元器件企业集聚发展，加快建成国家级铜基电子信息材料产业基地 完美世界小说。加快发展电子信息模具、塑料挤出模具和汽车模具等智能模具产业。加快推进高端农机和液压元件、武汉光谷黄石生物医药园、博天环保大冶产业园等项目建设，推动高端装备制造、生物医药、节能环保等产业发展，打造特色鲜明、竞争优势明显的产业集群。积极培育汽车零部件产业，力争成为省级重点成长性产业集群。

改造提升传统产业。实施新冶钢焦化节能环保改造、大冶有色20万吨废杂铜加工、山力兴冶薄板80万吨冷轧及镀锌、日丰新型建材等项目，不断提升资源能源利用效率和污染防治水平，促进工业绿色转型发展。实施光谷东机械产业园、奥莱斯轮胎二期、美尔雅工业园、力特隆30万锭特纺、劲牌健康产业园等一批重点项目，着力培育壮大接续替代产业。用好用活支持工业发展专项资金，支持企业技术改造、转型升级、做强品牌、增强竞争力。力争新增规模以上企业110家。

加快发展现代服务业。加快渤海鄂东商品交易中心、华中矿产品交易中心和长乐金融物流园建设，支持有色公司等企业服务业主辅分离，支持产权交易和股权托管交易市场发展，支持大型电商创新服务模式。大力发展文化创意和旅游业，支持文化传媒产业发展壮大，加强东方山景区和东方文化园建设，启动大众山森林公园建设，推进矿山公园整体托管开发，进一步挖掘乡村生态休闲旅游资源，提高旅游核心竞争力。积极培育消费热点，创新消费方式，鼓励网上消费，促进信用消费。进一步优化服务业布局，加快推进万达广场、尹家湖中心商务区等一批重点项目，建设服务业集聚区和示范区。推进武汉路、南京路、交通路等特色商业街发展。培育一批特色商贸镇。

加强自主创新能力建设。围绕战略性新兴产业，瞄准国际国内最高水平和最新标准，把互联网、云计算、大数据、物联网等新一代信息技术融入实体经济发展之中，切实增强自主创新能力。实施科技企业创业与培育工程，支持市级科技孵化器建设，新增高新技术企业10家以上、科技创业企业100家以上。加快科技对接武汉步伐，推动中科院黄石育成中心高效运行，促进企业创新平台提档升级，建设以企业为主体、市场为导向、产学研用紧密结合的技术创新体系。支持企业实施科技攻关项目。积极申报国家创新型试点城市。支持大冶开发区申报国家级高新区。深入实施质量强市战略，引导支持企业争创中国驰名商标、中国名牌和中国地理标志。

(三)全面深化改革开放，增强经济社会发展活力

深化行政领域改革。按市场化要求进一步转变政府职能、简政放权，制定政府权力清单、政府部门责任清单、企业投资项目负面清单，制定市县两级行政审批通用目录、公共资源交易项目基本目录、政府购买服务目录，依托政府网站完善政务信息共享平台。借鉴上海自贸区经验，支持市开发区和新港园区在行政审批等方面先行先试。围绕登记注册全程便利化，进一步完善“先照后证”、“三证联办”和企业信用信息公示制度，打造更加宽松的市场准入环境。

深化经济领域改革。严格执行新预算法，进一步完善预算管理。加大结转结余资金统筹力度，盘活财政存量资金。全面推进内贸流通体制改革，在营造法治化营商环境、“商旅文”融合发展等方面先行先试，趟出新路。加快推进多层次资本市场建设，实现企业上市和在股权托管交易中心挂牌3—4家。进一步扩大市中小企业担保中心资本金和融资规模，完善中小企业信用体系、融资担保工作机制，搭建银企合作平台，缓解中小企业融资难、融资贵。按照市场化、去行政化要求，深化市级投融资公司改革，整合经营性资产资源，支持市级投融资公司融资100亿元以上。积极探索政府与社会资本合作模式，支持基础设施和公共事业建设。按照“一企一策、股权多元化”要求，鼓励非公有资本、集体资本参与国有企业改革。继续处理好国企改制遗留问题。

深化农村改革。深入推进农村土地确权颁证工作。继续抓好工矿废弃地复垦利用工作，争取纳入国家新一轮试点。加强与武汉农村综合产权交易市场对接，积极推进农村综合产权交易体制改革。同时，统筹推进机关事业单位人事制度、全国和谐劳动关系综合试验区、社会保障、医药卫生体制等社会领域改革。

大力发展开放型经济。按照贸易便利化要求，复制上海自贸区经验，促进棋盘洲保税物流中心高效运行，对接武汉东湖综合保税区、东西湖保税物流中心，启动综合保税区申报工作。深化与盐田港集团合作，抓好棋盘洲港口堆场征地拆迁工作，确保港区7个泊位按约定时间投入运营，力争进港铁路、新港大道南延建成通车。深入实施“走出去”战略，搞好重点企业“一对一”帮扶，引导企业开拓新兴市场、开展境外投资和扩大境外工程承包。加强对外劳务服务中心建设，积极申报国家级外派劳务平台。加强区域合作，积极参与武汉城市圈有关试点工作，主动融入长江中游城市群一体化发展。

(四)坚持“三农”为重，努力在农业强、农民富、农村美上实现新突破

大力发展现代农业。全面落实各项强农惠农富农政策，稳定粮食产量。抓好大冶农产品加工区和阳新富河中游现代农业示范区建设，新建一批绿色农产品生产基地，进一步延长畜禽、现代渔业、食品饮料三个百亿元产业链，增强农副产品转化增值能力。加快培育新型农业经营主体，支持种养大户、家庭农场发展，建立农民合作社发展“绿色通道”，力争新型农业经营主体总量增长10以上。大力发展农产品加工业，新增省级龙头企业2家、规模以上农产品加工企业10家以上，农产品加工比值达到1.6以上。

着力改善农村生产条件。继续抓好民生水利工程建设，打通排灌渠道最后“一公里”，开工建设各类水利工程3100处，深化小型排灌设施管护体制改革。加快实施农村电网升级改造工程。实施整乡整村推进土地整治和复垦开发，加强高产农田建设，力争新建高标准基本农田4000公顷以上。加大扶贫攻坚力度，抓好32个重点贫困村、老区村和插花贫困村的脱贫解困工作。

加快美丽乡村建设。继续推进“四化同步”示范乡镇试点建设，加强主导产业培育，以产业发展带动人口集聚。坚持规划先行，按照重点建设中心村、科学保护特色村、全面整治培育村、搬迁撤并偏远村的思路，以“三万”活动为载体，突出抓好垃圾收集、卫生改厕、生活污水处理、村庄绿化等工作，创建美丽村庄25个。实施城乡保洁一体化工程，探索构建覆盖城乡的垃圾无害化收集处理体系，做好病死牲畜无害化处理工作，改善农村环境。

(五)着力推进新型城镇化，加快城乡一体化进程

全力推进大冶湖生态新区建设。深入推进新区16个重大基础设施项目，抓好核心区“五纵三横”道路建设，开工建设大冶湖两座跨湖大桥，加快推进黄石至阳新快速通道建设，开工建设筠山隧道，确保月亮山隧道贯通。全力推进省级园博园、生态展示馆、奥体中心、鄂东医疗中心等重点场馆和公共服务设施建设，以基础设施建设的大突破带动新区大发展。

着力完善城市功能。推进城乡交通设施建设，开工建设团城山客运枢纽站、阳新物流中心、棋盘洲长江大桥，做好武穴长江大桥建设前期工作，加快推进兴国至富池一级公路、316国道分线改造和武九客专大冶至阳新段建设，建成光谷大道、锦冶大道和阳新幕阜山连片扶贫开发旅游公路。完善城区基础设施，抓好苏州路、广州路、花湖大道延伸等重点工程建设和中心城区9处易渍水点改造，完成环磁湖区域排污口截流和东钢、老下陆、肖铺3条排洪港截污改造，完成河西污水处理厂、下陆新西港建设，加快西塞水厂一期、城区供水管网二期和城乡一体供水工程建设。新建天然气管网30公里，新增用户1.5万户。在中心城区建设过街通道1座，恢复社会停车场5处，新建公共停车场1处，取缔一批城市道路临时停车泊位，加强中心城区车辆违停整治，切实缓解交通拥堵。编制完成轨道交通规划和地下空间利用规划。加强测绘地理信息建设。加大旧城改造力度，加快实施人民街地块等22个城市棚改、垦区危改项目。实施城市管理疏导工程，按照市级统一规划、城区建设管理的原则，规划便民市场布点，合理设置农贸市场、小商品市场，实现摊点归市，全面化解流动摊贩、特殊市场占道经营等影响人居环境的问题。开展美好示范路创建工作。加快中心城区垃圾中转站建设。推进环卫作业市场化改革。

加快重点镇建设。启动编制黄石—大冶基础设施与公共设施同城化规划，以及西塞山区组团、铁山区组团、开发区汪仁章山组团总体规划，编制完成“华中康谷”及河口—黄颡口—富池、陈贵—灵乡—金牛、还地桥—保安—金山店城镇群的村庄体系规划，形成科学合理的城镇空间发展布局。加快推进重点镇、特色镇建设，打造灵乡、陈贵、王英3个省级重点镇和刘仁八、龙港2个省级特色镇。(六)坚持生态优先，持续改善人居环境

继续推进生态环境保护和治理。编制完成黄石基本生态线控制规划，适时划定生态红线。继续推进“五边三化”工程，重点抓好露天矿山关停和矿山生态修复、尾矿库综合治理，力争完成“五边三化”三年行动目标任务的75以上。完成柯尔山—白马山等生态公园建设，新增和改造绿地面积80公顷。加强工业领域重点行业污染防治，限期完成脱硫、除尘任务，淘汰黄标车5000辆，不断改善空气质量。编制完成水资源保护规划及水土保持规划。抓好劲牌公司毛铺和枫林酒厂生产水源地保护工作。加强以长江黄石段、富河和磁湖、大冶湖、保安湖、仙岛湖、网湖为重点的水环境保护与生态修复，加快实施磁湖等亚行水污染综合治理项目，完成青山湖综合治理一期和磁湖湿地公园一期工程。抓好农田土壤修复示范项目。

扎实推进节能减排。强化节能减排目标管理，全面完成主要污染物减排“十二五”目标任务。大力推进能源资源节约和循环利用，深入开展企业节能节水低碳行动。继续更新节能环保的新能源公交车辆，实施公交车“油改气”项目，确保清洁能源公交车占公交车总量的90左右 侯卫东官场笔记。全面落实建筑节能设计标准，新增绿色建筑12万平方米。狠抓主要污染物减排工作，围绕城镇污水处理厂及配套管网建设、工业领域重点行业污染防治、农业畜禽业污染源治理，实施一批减排项目。扎实推进国家循环经济示范城市创建、国家餐厨废弃物资源化利用和无害化处理试点、全国工业绿色转型发展试点和新能源示范城市建设工作。支持市开发区推进国家低碳工业园区试点工作。支持有色公司争创国家“城市矿产”示范基地。

深入推进各项创建工作。按照定目标、定任务、定责任的要求，统筹推进“五城同创”工作。成功创建4个省级生态村、20 个市级生态村。广泛开展文明单位、文明村镇、文明社区、文明家庭创建活动，力争在全国文明城市创建考评中取得好成绩。对照国家卫生城市创建新标准，制定三年行动方案，加大卫生社区创建力度，力争全市社区全部达到卫生社区创建标准，确保通过省级卫生城市复查和国家卫生城市省级考核。加快实施118个环保模范城创建项目，完成项目总数的60以上。扎实开展“绿满黄石”行动，新增植树造林15万亩。强化森林防火基层基础工作。认真抓好三级园博会筹办工作，按期完成中国(武汉)第十届国际园林博览会黄石展园建设和园区布展工作，成功举办第二届黄石(阳新)园博会。

(七)坚持民生为本，切实增进民生福祉

提升就业和社会保障水平。完善和落实创业就业扶持政策，深入实施科技孵化创业、大学生引领创业和农民工回归创业工程，切实抓好各级各类创业孵化基地、创业园(街)建设。抓好社会保险扩面征缴和清欠追缴，将被征地农民统一规范纳入养老保险范畴，力争“五险”参保新增2万人。全面落实职工生育保险和居民医疗保险新政策。进一步提高新农合保障能力，确保新农合参合率稳定在99以上。加大社会救助力度，提高城乡困难群众最低生活保障标准，全面建立“救急难”工作体系。加快推进养老服务社会化，完成市福利院新院一期工程，力争城市社区居家养老服务实现全覆盖，农村老年人互助式养老服务达到30。深入推进全国共有产权住房试点工作，完善住房供应体系，满足各类群体的住房需求。

提升基本公共服务水平。加快推进义务教育均衡发展，做好阳新县和市开发区省级评估验收工作。加大高中阶段学校布局调整力度，大力发展现代职业教育和民办教育，支持高校转型发展。完善和落实教育资助政策。积极推进教育信息化建设。继续推进公立医院改革，全面推行分级诊疗制度，巩固完善基本药物制度。深入开展乡镇卫生院、村卫生室、社区卫生服务中心提档升级活动。支持民营医疗机构发展。认真做好重大疾病防治、卫生应急和妇幼保健工作。依法治理出生人口性别比偏高和违法生育反弹问题，稳妥实施“单独二孩”政策。进一步完善公共文化设施，继续抓好农村文化室和文化礼堂建设。大力加强文艺创作，努力将话剧《刘伦堂》打造成精品剧目。加大文物保护力度，启动华新水泥厂旧址保护与展示利用工程，做好第一次全国可移动文物普查。加快全民健身公共服务体系建设，支持举办第二届半程马拉松邀请赛。启动省第十五届运动会筹备工作。

提升社会治理水平。加快推进全国社区治理和服务创新实验区建设，进一步抓好社区(村)网格化管理，加强社工队伍建设，建成20个城乡社区公共服务站。加强和改进信访工作，畅通群众诉求渠道，完善人民调解、行政调解、司法调解联动工作机制，及时化解各类矛盾纠纷。坚持不懈抓好安全生产，严格落实各级各方责任，持续抓好打非治违、超载超限专项整治，确保全市安全生产形势持续稳定。加强农产品、食品药品检测和安全治理能力建设，让人民群众吃上安全的食品、用上放心的药品。深入推进平安黄石建设，强化社会治安综合治理，加强立体化防控体系建设，严厉打击各类违法犯罪行为，保障人民群众安居乐业。

与此同时，编制完成“十三五”规划、2049年城市远景规划。加强全民国防教育，支持驻黄部队现代化建设，推动军民融合深度发展，争创省“双拥”模范城。全面贯彻落实党的民族宗教工作方针政策，继续抓好援藏、援疆等工作。深化与友好城市交流合作。重视和支持老龄事业发展。切实保障妇女和未成年人权益。继续做好统计、物价、史志、对台等工作。

今年，我们将认真办好人民群众关心的十件实事。(1)实现城镇新增就业5万人，就业创业培训1.5万人；帮扶成功创业6000人，带动就业1.8万人。(2)开工建设各类保障性住房19324套，基本建成9611套，分配13690套。(3)为困难群体和农民工办理法律援助案件1200件、法律援助事项12000件。(4)对150户贫困残疾人家庭实施无障碍改造，为300名0—6岁贫困残疾儿童提供抢救性康复服务，为贫困精神残疾人发放1000张服药卡，为贫困残疾人免费发放1500件辅助器具，免费为残疾人提供就业服务5000人次，为9000名特困重度残疾人提供生活补助。(5)对500例晚期血吸虫病人实施免费救治。(6)完成通村公路160公里。(7)创建30所义务教育阶段标准化学校，新建10所乡镇中心幼儿园，创建5所农村市级示范幼儿园。(8)实施“放心粮油”工程，建成30个粮油示范连锁店。(9)建设总长20公里城市绿道10条。(10)实施“黄石城镇光网改造工程”，新增光网覆盖家庭20万户，实现城市家庭光网全覆盖；实施广播“村村响”工程，覆盖3000个村组；实施有线网络智慧社区项目，建设完成1200个WIFI热点。

(八)转变政府职能，着力打造开明、精明、廉明、亲民政府

坚持依法行政。加强政府系统工作人员法治教育，增强遵守宪法和法律的自觉性，运用法治思维、法治方式推进工作，解决问题。自觉接受人大及其常委会的法律监督、工作监督，自觉接受人民政协的民主监督，认真办理人大代表意见建议和政协委员提案，进一步提高办结率。健全完善重大行政决策公众参与、专家论证、风险评估、合法性审查和集体讨论决定制度，建立重大决策终身责任追究、责任倒查机制，提高政府决策科学化、民主化水平。坚持依法行政，严格按照法定权限和程序履行职责、行使权力，构建“权界清晰、分工合理、权责一致、运转高效、法治保障”的行政职能体系，坚持做到严格规范公正文明执法。深化权力公开、决策公开、政务公开，完善政府信息发布制度，主动接受人民群众监督和舆论监督，让权力在阳光下运行。全面完成“六五”普法任务。

坚持高效行政。建立健全改进作风常态化机制，严格落实中央“八项规定”、省委“六条意见”和市委“六点要求”，坚决遏制“四风”，着力推进行政“不作为、慢作为、滥作为”等专项整治，不断巩固群众路线教育实践活动成果，以抓铁有痕的作风推动工作有效落实。强化行政效能监察，将政府目标考核管理全部纳入电子督查监察系统，实行项目化、目标化、时限化、责任化管理和全程跟踪问效。建立健全公务员“确责、履责、问责”责任体系，全面落实行政问责制，加强对滥用权力、行政缺位、决策失误、作风拖沓等问题的责任追究，打造一支清廉为官、事业有为的公务员队伍。建立完善以结果为导向的工作落实机制和常态化社会评议评价机制，健全政府重点工作定期通报、电视资政评议、群众代表询问质询制度，不断提升政府的执行力和公信力。

坚持廉洁从政。严格落实政府系统党风廉政建设责任制，全面加强惩治和预防腐败体系建设，深化岗位权力风险防控，加强公共资源交易监管，健全用制度管权、管事、管人长效机制，保证政府权力规范运行。坚持正风肃纪、猛药去疴，严厉查处违纪违法案件，坚决纠正行业不正之风，坚决查处损害群众利益行为，坚决严惩腐败分子，以廉政建设和反腐败斗争的新成效保障发展、取信于民。坚持俭朴从政、过紧日子，严格执行中央厉行节约、反对浪费的有关规定，继续开展“吃空饷”、“小金库”专项清理，严控“三公”经费支出，把更多的公共财力用于改善民生、推动发展。

各位代表!百舸争流，破浪者领航；千帆竞发，奋勇者当先。让我们在市委的坚强领导下，乘着全面深化改革的东风，适应新常态，干出新作为，为推动黄石转型发展作出新的更大的贡献!
